# Enhanced channel activity by PI(4,5)P_2_ ignites MLKL-related pathogenic processes

**DOI:** 10.1038/s41421-022-00451-w

**Published:** 2022-10-18

**Authors:** Bingqing Xia, Jingbo Qie, Xurui Shen, Sheng Wang, Zhaobing Gao

**Affiliations:** 1 State Key Laboratory of Drug Research, Shanghai Institute of Materia Medica, Chinese Academy of Sciences, Shanghai, China; 2 University of Chinese Academy of Sciences, Beijing, China; 3 Department of Immunology, School of Basic Medical Sciences, and Shanghai Pudong Hospital, Fudan University, Shanghai, China

**Keywords:** Necroptosis, Mechanisms of disease

Dear Editor,

Mixed lineage kinase domain-like protein (MLKL) emerged as executioner of necroptosis^1–3^. The intrinsic nature of MLKL and how it induces plasma membrane permeabilization, forming a huge pore or a channel, remain an interesting conundrum for a long time^4,5^. Our previous study demonstrated that MLKL forms cation channels and its channel activity is a primary effector of necroptosis^5,6^. In the field of MLKL, a major unsettled issue is, if MLKL does serve as channels, how it ignites the necrotic or non-necrotic pathogenic progresses^4,5^. Phosphatidylinositol 4,5-bisphosphate (PI(4,5)P_2_) is a necessary cofactor of various ion channels^7^. The physical interaction between MLKL and phosphatidylinositol phosphates (PIPs), including PI(4,5)P_2_, facilitates MLKL-mediated liposome leakage and the necrotic membrane disruption^8–10^. These studies have been directed toward understanding whether PI(4,5)P_2_ is a direct modulator for the MLKL channel activity. The potential effects of PI(4,5)P_2_ on MLKL channels were thus evaluated electrophysiologically and the subsequent pathogenic influences were explored.

The four-helical bundle domain (4HBD) in the N-terminal region of MLKL (MLKL^NT^) is sufficient to induce oligomerization and trigger cell death^9^. Consistent with our previous study, MLKL^NT^ protein exhibited channel activity, translocated onto plasma membrane, and caused cell death, similarly to full-length protein (MLKL^FL^) (Supplementary Figs. S1 and S2). We next asked whether lipids could regulate the channel activity. Interestingly, we found that the typical single-channel currents could only be recorded when the phosphatidylcholine (PC) bilayers were premixed with phosphatidylserine (PS). Different from the neutrally charged PC, PS is a type of negatively charged phospholipid (Fig. 1a; Supplementary Fig. S3a, b). Afterward, MLKL channel activity was tested with some other important negatively charged phospholipids. Under the identical experimental conditions, we observed much larger step-like signals in the 3PC/2PS lipids premixed with 2% PI(4,5)P_2_ compared with those without PI(4,5)P_2_ (Fig. 1a). The overall open probability (*Po*) was enhanced to 27.4% (Fig. 1b). These step-like currents exhibited two conductance states (Fig. 1b, c; Supplementary Fig. S3c). Influences of other lipids on MLKL channel activity were tested, including phosphatidylinositol 3,4-bisphosphate (PI(3,4)P_2_) and phosphatidylinositol 3,5-bisphosphate (PI(3,5)P_2_), phosphatidylinositol 4-phosphate (PI(4)P), 1,4,5-trisphosphate inositol (IP_3_) and diacylglycerol (DAG), phosphatidylinositol 3,4,5-trisphosphate (PIP_3_)^11^. Among these lipids, PI(4)P is the only one that increases MLKL channel open probability (Fig. 1b; Supplementary Fig. S3a, b). Collectively, these data indicate that the anionic phospholipids are essential for the channel function and the channel activity could be modulated by the PIPs, particularly PI(4,5)P_2_. Subsequently, we further tested MLKL channel activity in the presence of different concentrations of PI(4,5)P_2_. An increased concentration of PI(4,5)P_2_ induced more frequent and larger currents of MLKL channel (Fig. 1c, d; Supplementary Fig. S4a, b). To further evaluate whether the observed influences were due to the changes in PI(4,5)P_2_ level, a well-recognized approach was exploited to manipulate PI(4,5)P_2_ levels in live cells. Stimulation of the M1 muscarinic receptor can activate PLC-β which hydrolyzes PI(4,5)P_2_. PLC-β-PH-GFP (PLC-PH) construct, a PI(4,5)P_2_ reporter, is used to monitor the PI(4,5)P_2_ distribution on plasma membrane^12^. Here, MLKL, M1 receptor, and PLC-PH were co-transfected into HEK293 cells. With 5 μM oxotremorine M (Oxo-M) treatment, the PLC-PH probe dissociated from the plasma membrane to cytoplasm, showing PI(4,5)P_2_ hydrolyzation (Fig. 1e). Meanwhile, MLKL currents were rapidly inhibited and restored after Oxo-M withdrawal (Fig. 1f, g; Supplementary Fig. S4c). These data indicate that MLKL channel activity could be finely tuned by PI(4,5)P_2_.

**Fig. 1:**
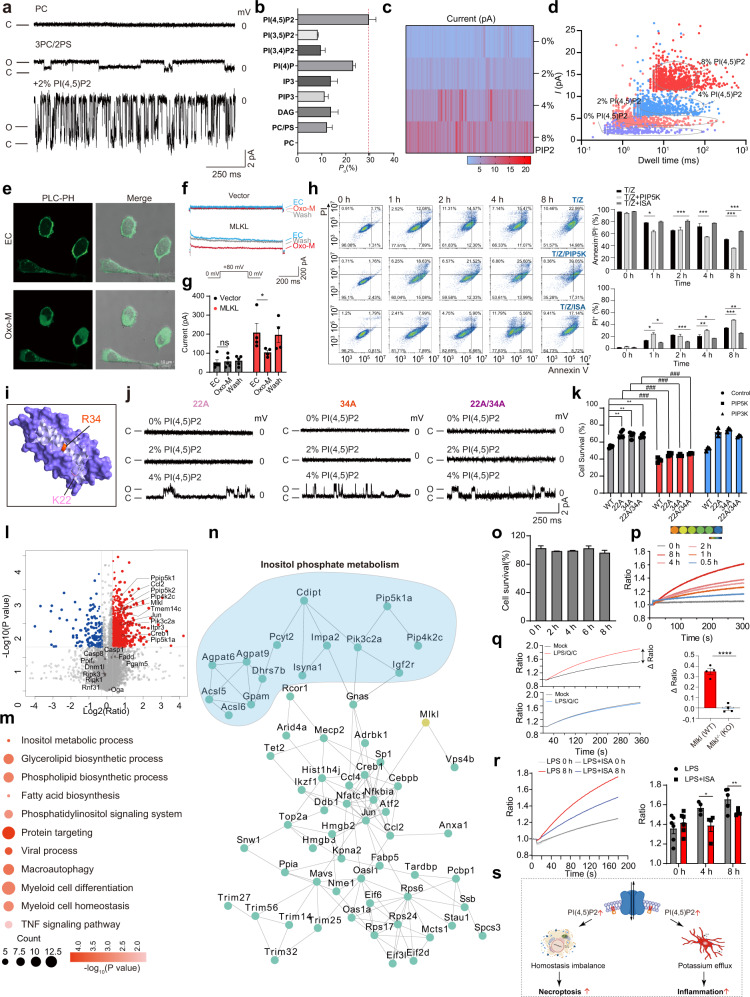
Enhanced MLKL channel activity by PI(4,5)P_2_ promotes necroptosis and inflammation. **a**, **b** Single-channel current recordings and open probability of MLKL^NT^ in different lipid compositions (*n* > 3). **c** The heatmap shows MLKL^NT^ currents in various PI(4,5)P_2_ concentrations. **d** Scatterplots of MLKL^NT^ currents vs. dwell times. **e** Representative subcellular localization of GFP-PH domain. Cells were treated with or without 5 μM Oxo-M. **f** The currents of MLKL channel with or without Oxo-M treatment. EC indicate the extracellular solution. **g** Statistical analysis of MLKL currents (*n* ≥ 3). **h** Necroptosis was detected through Annexin V-FITC (AV)/PI staining. L929 cells were treated with 20 ng/mL T and 20 μM Z after PIP5K transfection or ISA treatment (left). Statistical analysis of AV/PI staining (right). **i** Schematic representation of the residues examined by the solution MLKL^NT^ structure. **j** Current traces of MLKL mutants 22A, 34A, and 22A/34A in the presence of different concentrations of PI(4,5)P_2_. **k** Cell viability of MLKL^–/–^ HeLa cells after co-transfection with PIP5K or PIP3K and MLKL mutant constructs. **l** The volcano plots for the comparison between proteome patterns of BV2 cells treated with PBS or LPS. **m** Representative function enrichment analysis of upregulated DEGs. Functional terms were labeled and color-coded with log_10_ transformed *P*-value (Fisher’s exact test). **n** PPI network of proteins shown in Supplementary Fig. S8. Proteins participating in inositol phosphate metabolism are labeled with blue. **o** Cell viability of BV2 cells after LPS treatment. **p**, **q** Potassium efflux levels in LPS-treated BV2 cells and LPS/Q/C-treated BMDM cells (left). Statistical analysis results were shown (right). **r** Detection of K^+^ efflux level of LPS-treated BV2 cells with or without ISA (left). Statistical analysis results were shown (right). **s** Enhanced MLKL channel activity by PI(4,5)P_2_ promoted necroptosis and inflammation via disturbing ion homeostasis and accelerating potassium efflux.

Whether the PI(4,5)P_2_-induced augmentation of MLKL channel activity is correlated with cell death was examined. To elevate PI(4,5)P_2_ concentrations in the plasma membrane, PIP5K, one of the key PI(4,5)P_2_ biosynthetic enzymes, was transfected into L929 cells (Supplementary Fig. S5). A time-dependent increase in the number of fluorescent cells stained by Annexin V-FITC and prodidium iodide (PI) was observed in PIP5K-transfected L929 cells compared to non-transfected cells with tumor necrosis factor (TNF-α, T) plus pan-caspase inhibitor z-VAD-fmk (Z) treatment^1^ (Fig. 1h; Supplementary Fig. S6a). In addition, we found that either knockdown of PIP5K or administration of the PIP5K inhibitor ISA-2011B (ISA) efficiently suppressed the TNF-induced necroptosis in L929 cells (Fig. 1h; Supplementary Fig. S6b). Similar PI(4,5)P_2_ influences were observed in HeLa cells (Supplementary Fig. S6c, d). PIP3K was used as a negative control (Supplementary Fig. S5). Collectively, these results support that the elevated PI(4,5)P_2_ level aggravated MLKL-dependent necroptosis.

For many PI(4,5)P_2_-sensitive ion channels, PI(4,5)P_2_ binds with positive amino acid via “electrostatic interaction”^7,13^. We mutated the positive amino acids of MLKL^NT^ to alanine and tested the channel activity (Supplementary Fig. S7a–c). Two of these mutations, K22A (22A) and R34A (34A), were found to abolish the channel function regardless with or without 2% PI(4,5)P_2_ (Fig. 1i, j). Notably, the channel functions were rescued under higher PI(4,5)P_2_ concentrations (4%) (Fig. 1j; Supplementary Fig. S7d). Whether these mutant channels could lead to cell death was then investigated. In comparison to WT MLKL^NT^, the capability of killing cells of the two mutant channels was lost or largely suppressed in MLKL^–/–^ HeLa cells^9^ (Fig. 1k). Consistently, the two mutants became toxic to cells after overexpression of PIP5K that elevated PI(4,5)P_2_ levels on membranes. Of note, the double mutant K22A/R34A did not further reduce the PI(4,5)P_2_ sensitivity or abrogate the capability of killing cells after overexpression of PIP5K, suggesting that K22 and R34 may interact with PI(4,5)P_2_ independently (Fig. 1j, k).

MLKL could activate the innate immune receptor nucleotide-binding oligomerization domain (NOD)-like receptor protein 3 (NLRP3) in a cell-intrinsic manner before cell lysis, but its working model has not yet been clarified^14^. Thus, we further explored the potential function of PI(4,5)P_2_-enhanced MLKL channel activity in an inflammation model^12^ (Supplementary Table S1). The proteome profiling of mouse microglia (BV2 cells) treated with lipopolysaccharide (LPS) showed that expression of traditional MLKL-binding proteins related to cell death (such as RIPK1, RIPK3) was not influenced by LPS, indicating that this inflammatory process was independent of classical necroptosis pathway (Fig. 1l). Gene enrichment analysis on differentially expressed genes (DEGs) showed activation of inositol metabolism and phospholipid metabolic processes, as well as inflammatory responses induced by LPS treatment (Fig. 1m; Supplementary Fig. S8). Of particular relevance is that enzymes responsible for PI(4,5)P_2_ anabolism were upregulated by LPS treatment, along with the LPS-induced inflammation-related proteins (Fig. 1n; Supplementary Fig. S8). To investigate the molecular regulation network of the DEGs related to PI(4,5)P_2_ metabolism and inflammatory responses, we searched their protein–protein interactions (PPIs) through String database (https://string-db.org), and further revealed the close relationship between PI(4,5)P_2_, MLKL and LPS-induced inflammation (Fig. 1n).

Consistent with proteomic data, treatment with the lower concentration (10 ng/mL) of LPS significantly stimulated the secretion of inflammatory cytokines, but did not induce cell death, while the expression level of MLKL and PIP5K increased (Fig. 1o; Supplementary Fig. S9a, b). Accordingly, an essential role of MLKL-dependent K^+^ efflux in triggering inflammation has been proposed^14,15^. We therefore hypothesized that K^+^ efflux during inflammation is mediated by MLKL channels. A real-time, sensitive FluxOR™ assay was performed to monitor the K^+^ efflux during the LPS-induced inflammation. We found that the K^+^ efflux gradually increased in a time-dependent manner (Fig. 1p). The contribution of MLKL channel to the K^+^ efflux was further evaluated in a widely used MLKL-related inflammation model, bone marrow-derived macrophages (BMDMs) treated with LPS, Smac-mimetic compound (C) and a caspase inhibitor Q-VD-OPh (Q)^14^. Both the MLKL-mediated cytokine secretion and the LPS-induced K^+^ efflux were abrogated in MLKL-knockout (MLKL^–/–^) cells (Fig. 1q; Supplementary Fig. S9c, d). Whether inhibition of PI(4,5)P_2_ synthesis after ISA treatment suppresses K^+^ efflux was examined in two inflammation models. After administration of ISA, the K^+^ efflux was indeed decreased in both the LPS-induced and the classical NLRP3 activation inflammation models (Fig. 1r; Supplementary Fig. S9f). Since elevated PI(4,5)P_2_ level is a key factor for the MLKL-related inflammation, we used PIP5K-knockdown BV2 cells to detect cytokine secretion level (Supplementary Fig. S9g). The result showed that blocking the PI(4,5)P_2_ synthesis indeed suppressed the secretion of inflammatory cytokines (Supplementary Fig. S9h). These results link PI(4,5)P_2_ with K^+^ efflux in MLKL-related inflammation.

In summary, we show that MLKL channel activity is fine-tuned by PI(4,5)P_2_ in a dose-dependent manner for the first time (Fig. 1c–g). PI(4,5)P_2_ exhibits agonistic effects on MLKL channel activity and the enhanced channel activity, alone or with other factors together, may ignite necroptosis or inflammation under specific stimuli (Fig. 1s). Whether and how the fine-tuned MLKL channel activity by PI(4,5)P_2_ participates in more substantial biological functions of MLKL, such as immune escape, nerve regeneration and vesicle transport, warrants further investigation.

## Supplementary information

Supplementary information

Supplementary Tabel S1

## Data Availability

All proteome data generated in this study, including the raw files and quantitative data matrix of proteomes, have been deposited to PRIDE (https://www.ebi.ac.uk/pride) with accession number PXD030814 (and can be accessed with account: reviewer_pxd030814@ebi.ac.uk; Password: tLngFikB).
