# Supplementary material for: Enhanced channel activity by PI(4,5)P2 ignites MLKL-related pathogenic processes
Source: Cell Discov. 2022 Oct 18;8:111. doi: 10.1038/s41421-022-00451-w (PMC9576727; doi:10.1038/s41421-022-00451-w)
Supplement: Supplementary file 1 — Supplementary information [file 41421_2022_451_MOESM1_ESM.pdf]

Supplementary information

Materials and Methods

Protein purification and mutagenesis

Human MLKL^{NT} and MLKL^{CT} proteins were purified using glutathione-Sepharose affinity chromatography (Yeasen) as previously published[1]. The mutant sequence was generated by site-directed mutagenesis and confirmed by sequencing (BGI, China).

Planar lipid bilayers recording

The purified proteins were incorporated into lipid bilayers to test their functionality. All the lipids were bought from Avanti (Avanti Polar Lipids, USA). Proteins were added to *cis* side and single channel currents were recorded under voltage-clamp mode using a Warnner bilayer clamp amplifier BC-535 (Warner Instruments, USA), filtered at 1–2 kHz. The recording frequency was 10 kHz. The currents were digitized using pClamp 10.2 software (Molecular Devices, US). Data are presented as means ± SEM. The single-channel conductance and open time were determined by fitting to Gaussian functions (bin width = 0.125 pA) or to single or bi-exponential equations. Opening times less than 0.5–1.5 ms were ignored. The equilibrium potential was calculated using the Nernst equation and Goldman-Hodgkin-Katz flux equation.

Whole-cell patch clamp recordings

Whole-cell channel currents were recorded using an Axopatch 200B amplifier. Data were digitized at 10 kHz and digitally filtered at 1 kHz. Patch electrodes were pulled from borosilicate glass and fire-polished to a resistance of ~3 MΩ when filled with internal solutions. The extracellular solutions contained NaCl 140 mM, KCl 5 mM, $MgCl_2$ 1.25 mM, $CaCl_2$ 1 mM, glucose 10 mM and HEPES 10 mM (adjusted to pH=7.4 with NaOH). The internal solution contained CsCl 140 mM, $MgCl_2$ 1 mM, $CaCl_2$ 1 mM, HEPES 10 mM and EGTA 10 mM (adjusted to pH=7.2 with KOH). All experiments were conducted at room temperature (23–26 °C), and all recordings were analyzed using pCLAMP10.6.

Cell culture and treatment

L929, HEK293, BV2, HeLa and HeLa MLKL KO cells were grown in 90% DMEM basal medium (Gibco, USA) supplemented with 10% fetal bovine serum (Gibco, USA). BMDM cells were grown in 90% DMEM basal medium (Gibco, USA) supplemented with 10% fetal bovine serum (Gibco, USA) and 25 ng/mL mouse M-CSF (416-ML-010, R&D, USA). All cells were cultured under 5% CO_2 at 37 °C. L929 HEK293, BV2 and HeLa cells were purchased from National Collection of Authenticated Cell Cultures (China). HeLa MLKL KO cells were obtained as a gift from Prof. Huayi Wang (Shanghai Tech University, China). MLKL KO BMDMs were isolated from MLKL-knockout mice, which was obtained as a gift from Prof. Xin Xie (CAS Key Laboratory of Receptor Research, Shanghai Institute of Materia Medica, Chinese Academy of

Sciences). The lentivirus for sh-PIP5K (hU6-MCS-CBh-gcGFP-IRES-Puromycin) knockdown was purchased form GENE company (GENE, China). Compounds were TNF-α (10602-HNAE, SinoBiological, China), ISA-2011B (HY-16937, MedChemExpress, USA) pan-caspase inhibitor z-VAD-fmk (HY-16658, MedChemExpress, USA), BV6 (HY-16701, MedChemExpress, USA) and Oxotremorine M (1067, Tocris Bioscience, USA), nigericin (HY-127019, MedChemExpress, USA). L929 cells were treated with 20 ng/mL TNF-α plus 20 μM zVAD-fmk (TZ) and HeLa cells were treated with 20 ng/mL TNF-α, 100 nM BV6 plus 20 μM zVAD-fmk (TSZ) to establish necroptosis models. To detect the effect of MLKL channel on inflammation, BV2 cells were treated with 10 ng/mL LPS for different times or treated with 10 ng/mL LPS for 4 h followed by 10 μM nigericin for 2 h (LN).

Western blotting and antibodies

Proteins were resolved in 12% SDS-PAGE, transferred to PVDF membranes (GE), and incubated with primary antibodies against mouse MLKL (AP14272B, Abcepta, China), p-MLKL (Ser345) (37333, CST, USA), PIP5K (NBP2-19833, Novus, USA), NLRP3 (15101, CST, USA) and GAPDH (30201ES60, Yeasen, China). Second antibodies are peroxidase-Conjugated Goat Anti-Rabbit IgG (H+L) (33101ES60, Yeasen, China) and Peroxidase AffiniPure Goat Anti-Mouse IgG (H+L) (33201ES60, Yeasen, China).

Immunofluorescence and imaging

To detect the localization of MLKL, HEK293 or HeLa cells were seeded as 1.5×10^5 cells per well in the 6-well plates (30720113, Thermo Fisher Scientific Inc., US). After transfection for 24 h, cells were fixed by 4% PFA, followed by staining with DiI/DiO (plasma membrane dyes, 40726ES10, 40725ES10, Yeasen, China) and DAPI (40728ES03, Yeasen, China). Cells were later captured using Olympus FV 1000 confocal microscopy (Olympus, Japan) with 100× oil objective. All image data shown are representative of at least three randomly selected fields.

To detect the level of $PI(4,5)P_2$, L929 cells were co-transfected with PIP5K and GFP plasmids. PIP3K were used as negative control and GFP indicated the transfected cells. After 16 h transfection, the cells were fixed with 4% PFA for 10 min at room temperature, followed by 0.2% Triton X-100 permeabilization for 20 min. After 3 times washes by PBS, the cells were then blocked for 30 min in blocking buffer containing 2% BSA in PBS. Later, the cells were immunolabeled at room temperature for 2 h with the primary $PI(4,5)P_2$ antibody (Abcam, ab11039) in blocking buffer. After being washed by PBS for 3 times, cells were incubated with a Cy3-conjugated secondary antibody (Yeasen, 33208ES60) for 2 h at room temperature followed by 3 times washes with PBS. The nuclei were stained with DAPI (Yeasen, 40728ES03). Finally, we mounted coverslip with anti-fluorescence quenching mounting medium (Yeasen, 36307ES08) and observed fluorescence under laser scanning confocal microscope (Leica TCS-SP8 STED). DAPI was observed in 405 nm channel, GFP was observed in

488 nm channel, Cy3 indicated $PI(4,5)P_2$ was observed in 552 nm channel by 60× oil object. Statistical analysis was performed using Fuji software.

Flow cytometry of cell death

After treatment, cells were collected by centrifugation and resuspended in 1× binding buffer containing 100 μg/mL propidium iodide (PI) and Annexin V-FITC (1:20, V13242, Life, USA). Cells were analyzed by flow cytometry using CytoFlex (USA, Beckman Coulter). The percentages of differently labeled cells were calculated by CytExpert software.

ELISA

Cell culture supernatants and serum were assayed for mouse IL-6 (VAL604, R&D Minnesota, USA), mouse TNF-α (VAL609, R&D Minnesota, USA) according to the manufacturer's instructions.

Cell viability

Cell viability were tested using the CCK-8 kit (40203ES60, Yeasen, China). The absorbance was measured at 450 nm with Thermo Scientific Microplate Reader (Thermo Fisher Scientific Inc., USA).

FluxOR reagents and assay

Potassium measurements were performed by FluxOR™ Potassium Ion Channel Assay (F10017, life, USA). All the procedure followed the manufacturer's instructions. Briefly, BV2 cells were seeded in 96-well plate (655090, Greiner Bio-One, Germany) for 10,000 cells per well. After LPS treatment for the indicated times with or without ISA-2011B, cells were loaded with loading buffer (80 μL/well) and incubated at room temperature in the dark for 60 min to load the ion dye. Then, the loading buffer was replaced with assay buffer. Cells were measured with a FDSS 7000 kinetic plate reader (Hamamatsu, Hamamatsu City, Japan) in 1-s intervals for 180 s using a standard Fluo-4 480-nm excitation and 530-nm emission filter set. A baseline recording of 10 cycles was recorded prior to stimulation.

Sample preparation for proteome

For each treatment group, microglia in mouse brain were collected and washed in PBS and subjected to global protein extraction using 8 M Urea (PH 8.0) containing protease inhibitor (phenylmethanesulfonyl fluoride, PMSF), followed by 3 min of sonication (3 s on, 3 s off, amplitude 25%). Then the protein concentration was obtained through Bradford quantification assay and 100 μg protein was digested overnight following filter-acid sample preparation (FASP) method[2] with 3.5 μg trypsin in 50 mM ammonium acid carbonate (PH 8.0) overnight at 37 °C. Finally, the purified peptides were acquired after extraction with 50% acetonitrile (ACN) and 0.1% formic acid (FA)

following desalination in two layers of Empore 3M C18 disk with 2 mg packing (3 μm, 150 Å, Agela) in a pipet tip and dried in a vacuum concentrator (Thermo Scientific).

Liquid chromatography tandem mass spectrometry (LC-MS/MS) analysis of peptide mixture

Proteome analysis were processed on a nanoElute-HPLC System (Bruker Daltonics) coupled with a hybrid trapped ion mobility spectrometry quadrupole times-of-flight mass spectrometer (TIMS-TOF Pro Bruker Daltonics, Billerica, MA) via a Captive Spray nano-electrospray ion source. Peptide mixture were re-dissolved in solution A (0.1% FA) and loaded onto the analytical column (75 μm i.d. × 25 cm) and separated with a 60 min gradient (2%−22% solvent B (ACN with 0.1% formic acid) for 45 min, 22%−37% B for 5 min, 37%−80% B for 5 min, and then 80% B for 5 min) at a flow rate of 600 nL/min. The MS analysis was performed by scanning 100–1700 m/z in positive electrospray mode. The accumulation and ramp time were set as 100 ms each. Survey full-scan MS spectra (100–1700 m/z) were obtained. The ion mobility was scanned from 0.7 to 1.3 Vs/cm^2. The overall acquisition cycle of 1.16 s comprised one full TIMSMS scan and 10 parallel accumulation-serial fragmentation (PASEF) MS/MS scans. During PASEF MSMS scanning, the collision energy was ramped linearly as a function of the mobility from 59 eV at $1/K0 = 1.6\ Vs/cm^2$ to 20 eV at $1/K0 = 0.6\ Vs/cm^2$.

Proteome identification and quantification with MaxQuant-based database searching

MS raw files were searched against the Swiss-Prot database (downloaded on August 20, 2020, containing 20,375 protein sequence entries) using PEAKS Online Xpro Software (v1.4) for peptide and protein identifications. Trypsin was selected as the proteolytic enzyme, and three missed cleavages sites were allowed. The mass tolerance was 15 ppm for precursor and 0.05 Da for production. The oxidation of Methionine and N-acetylation were set as the variable modifications. The false discovery rates of the peptide-spectrum matches (PSMs) and proteins were set to < 1%. For the proteome quantification, the area values under the curve (AUC) of a peptide feature were subjected to further analysis.

Function enrichment analysis

Proteins with significant changes ($P < 0.05$, bilateral Student's *t*-test) are colored with blue (fold change ≤ 0.5) or red (|fold change| ≥ 1.2) (Fig. 1i). Gene function enrichment analysis was performed with the R Bioconductor package 'clusterProfiler' (R package v3.14.3) based on Gene Ontology (GO) database or Kyoto Encyclopedia of Genes and Genomes (KEGG) database. Enrichment scores were determined using Fisher's exact test. The protein-protein networks (PPIs) were explored through STRING database and visualized using cytoscape 3.8.0.

Related references

1 Ying, Z. *et al.* Mixed Lineage Kinase Domain-like Protein MLKL Breaks Down Myelin following Nerve Injury. *Mol Cell* **72**, 457-468.e455 (2018).

2 Wisniewski, J. R., Zougman, A., Nagaraj, N. & Mann, M. Universal sample preparation method for proteome analysis. *Nature Methods* **6**, 359-362 (2009).

Supplementary Figures and Table

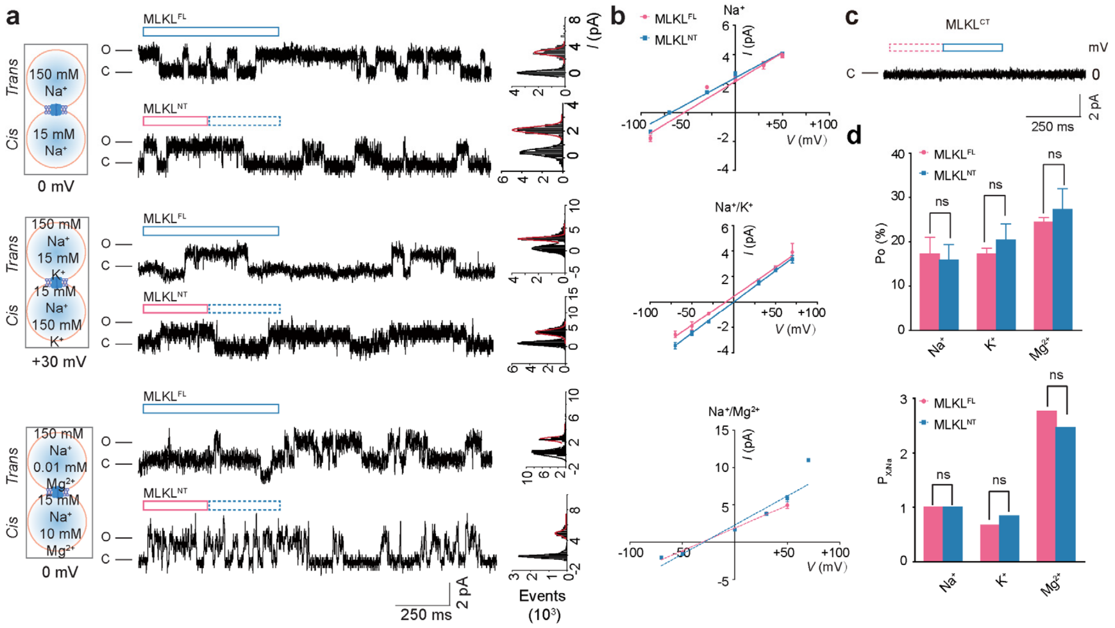

Supplementary Figure S1 Comparison of MLKL^{NT} and MLKL^{FL} protein channel activity parameters in planar lipid bilayers. a Single-channel current recordings of the MLKL^{NT} and MLKL^{FL} after reconstitution in lipid bilayers with the indicated solutions at 0 mV. Protein (5-50 ng/mL) was added to the *cis* side. Representative amplitude histograms from left trace are shown. **b** *I-V* curves of MLKL^{NT} and MLKL^{FL} proteins in different solutions as indicated in **a**. **c** C-terminal region of MLKL protein (MLKL^{CT}, 3.0 μg/mL) did not induce step-like current signals in sodium solutions. **d** Open probability and ion selectivity comparison between MLKL^{NT} and MLKL^{FL} in different solutions as indicated in **a**.

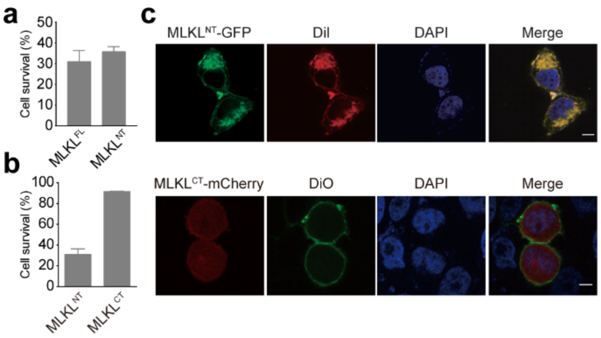

Supplementary Figure S2 MLKL^{NT} is sufficient to induce cell death and locates on plasma membrane. a Cell survival rate of HeLa$^{-/-}$ cell after transfection with MLKL^{FL} and MLKL^{NT} plasmids, respectively. **b** Comparison of the ability of MLKL^{NT} and MLKL^{CT} in inducing cell death. **c** Images of HeLa$^{-/-}$ cells after transfection of MLKL^{NT}-GFP (upper) or MLKL^{CT}-mCherry (lower) by confocal microscopy (DiI/DiO, membrane marker; DAPI, nuclear marker). Scale bars, 10 μm.

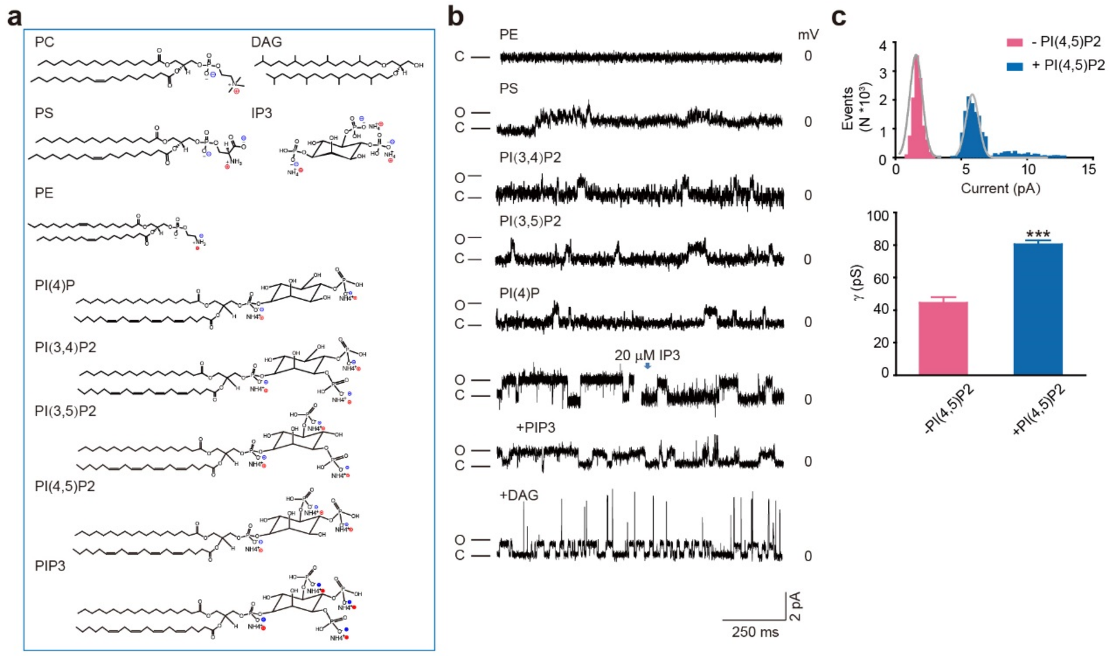

Supplementary Figure S3 Channel activity of MLKL^{NT} in planar lipid bilayers of different lipid compositions. a Chemical structures of the phospholipids used in this study. **b** Single-channel currents of MLKL^{NT} were recorded in potassium solutions under indicated membrane lipids. **c** Top, All-point amplitude histogram generated from the continuous single-channel recording of a traces (PC/PS=3/2 with or without PI(4,5)P_2). The two peaks represent the current levels of the single channel with or without PI(4,5)P_2. The line represents a Gaussian fit to the binned data (bin width = 0.125 pA). Bottom, conductance of MLKL^{NT} with or without PI(4,5)P_2 ($n > 6$).

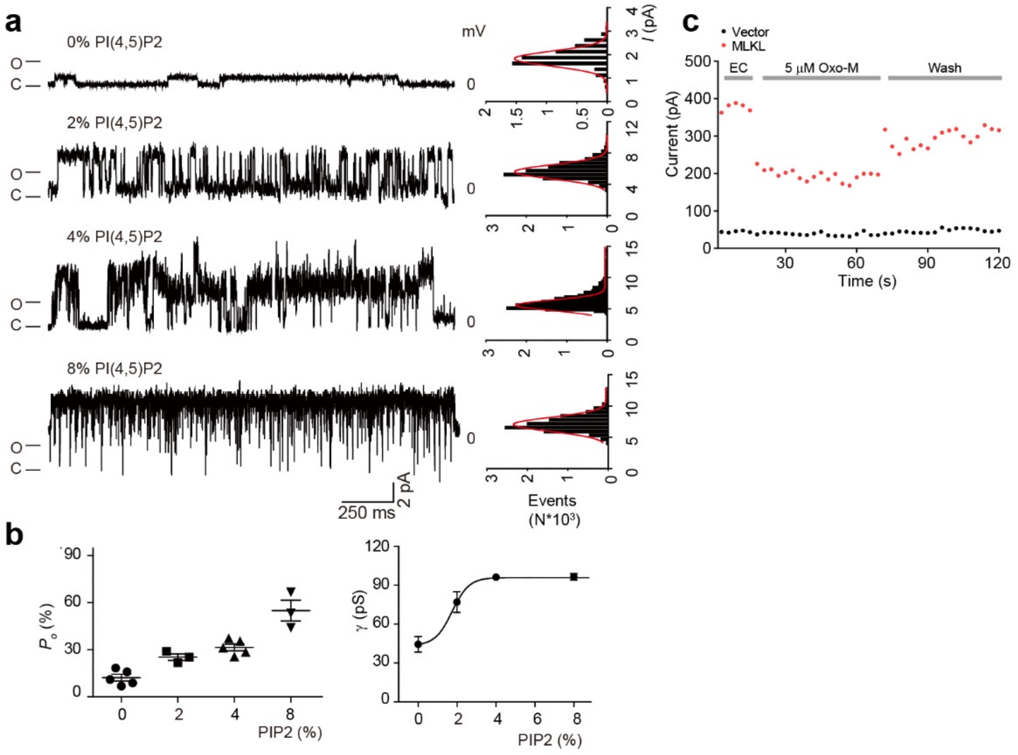

Supplementary Figure S4 Concentration dependence of the PI(4,5)P_2 influence on MLKL^{NT} channels. a Representative single-channel current recordings (Left) and all-point current histograms (Right) of MLKL^{NT} in the presence of various concentrations of PI(4,5)P_2. **b** Open probability (Left) and conductance (Right) of MLKL^{NT} in the presence of various concentrations of PI(4,5)P_2. **c** Kinetics of MLKL current inhibition after Oxo-M treatment.

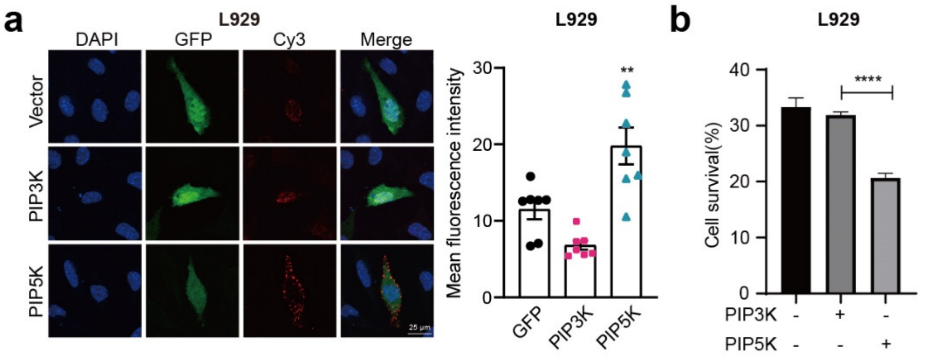

Supplementary Figure S5 PI(4,5)P_2 expression and cell survival level after PIP5K overexpression in L929 cells. a Expression level of PI(4,5)P_2 in L929 cells by immunofluorescence (DAPI, nuclear; GFP, the transfected cell; Cy3, PI(4,5)P_2) (Left). Analysis of the Cy3 fluorescence intensity was shown (Right). **b** Cell viability of L929 cells after PIP5K or PIP3K transfection and 20 ng/mL TNF-α and 20 μM pan-caspase inhibitor z-VAD-fmk (TZ) stimulation.

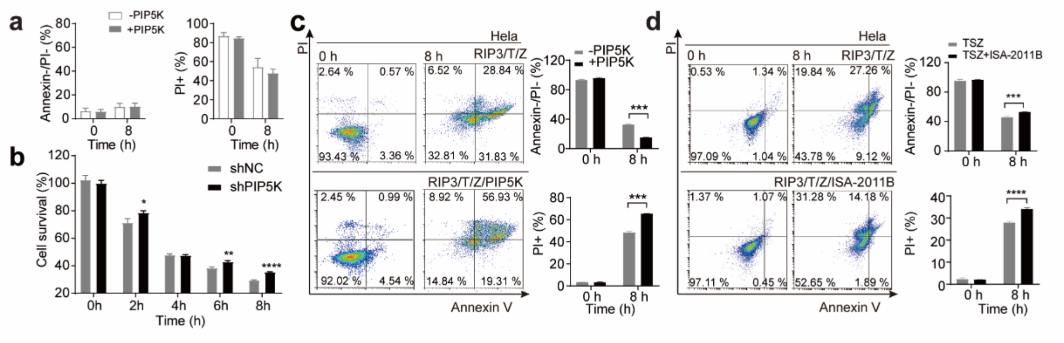

Supplementary Figure S6 PI(4,5)P$_2$ facilitates MLKL-dependent necroptosis. a Overexpression of PIP5K in L929 cells did not induce cell death. **b** L929 cells showed downregulated PI(4,5)P$_2$ levels by PIP5K knockdown and were treated with 20 ng/mL TNF-α (T) plus 20 μM z-VAD-fmk (Z). The cell viability was detected by CCK8. **c** HeLa cells were treated with 20 ng/mL TNF-α (T) plus 20 μM z-VAD-fmk (Z) and 10 nM BV6 (S) (T/S/Z) after transfection with RIPK3 in the presence or absence of PIP5K. **d** HeLa cells were treated with T/S/Z (TNF-α, z-VAD-fmk and BV6) after transfection with RIPK3 in the presence or absence of PIP5K inhibitor ISA-2011B. Necroptosis was detected using Annexin V-FITC/PI staining and analyzed by flow cytometry. **P* < 0.05; ***P* < 0.01; ****P* < 0.001; *****P* < 0.0001; unpaired Student's *t*-test. All error bars are SEM.

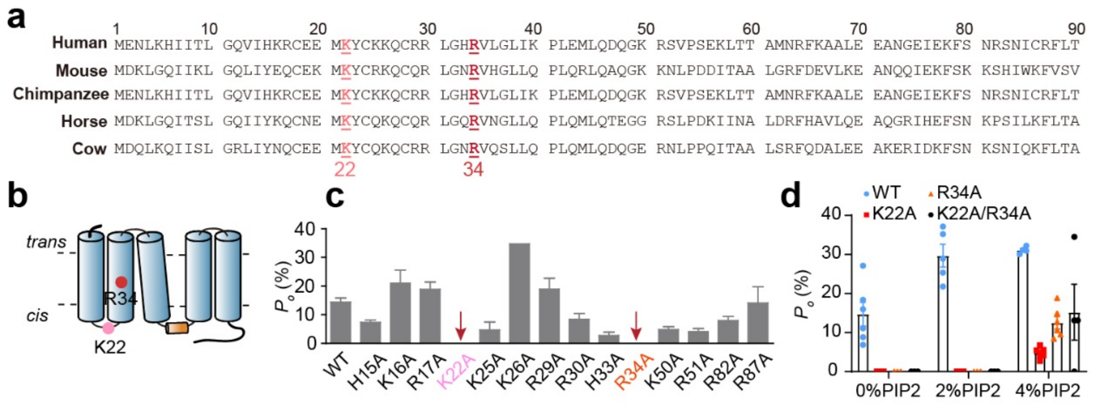

Supplementary Figure S7 MLKL^{NT} schematic representation, mutation channels open probability and cell survival. a Illustration of the amino-acid sequence of MLKL (1-90) and the indicated mutants. **b** Schematic representation of the residues examined by the solution MLKL^{NT} structure. **c** Open probability for MLKL^{NT} mutant channels in 2% PI(4,5)P_2 under 0 mV (n > 3)(Right). **d** Open probability under various PI(4,5)P_2 concentrations.

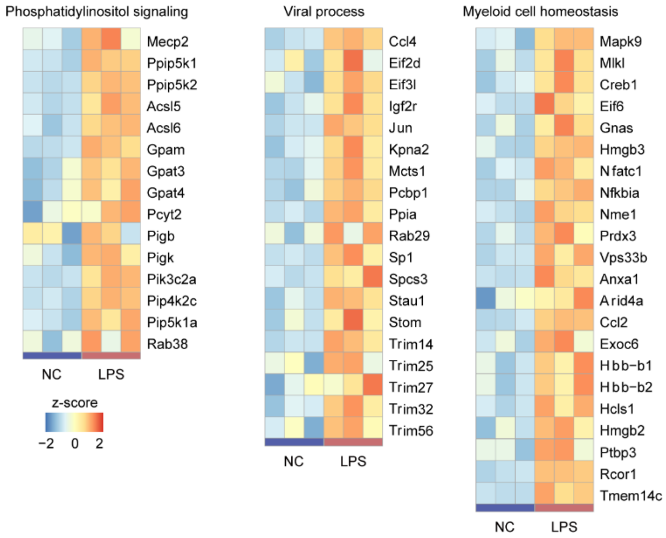

Supplementary Figure S8 Expression patterns of proteins participating in the indicated cellular functions/pathways among BV2 cells treated with PBS or LPS for 8 h. Values for each protein in all groups are color-coded based on the z-scored expression values.

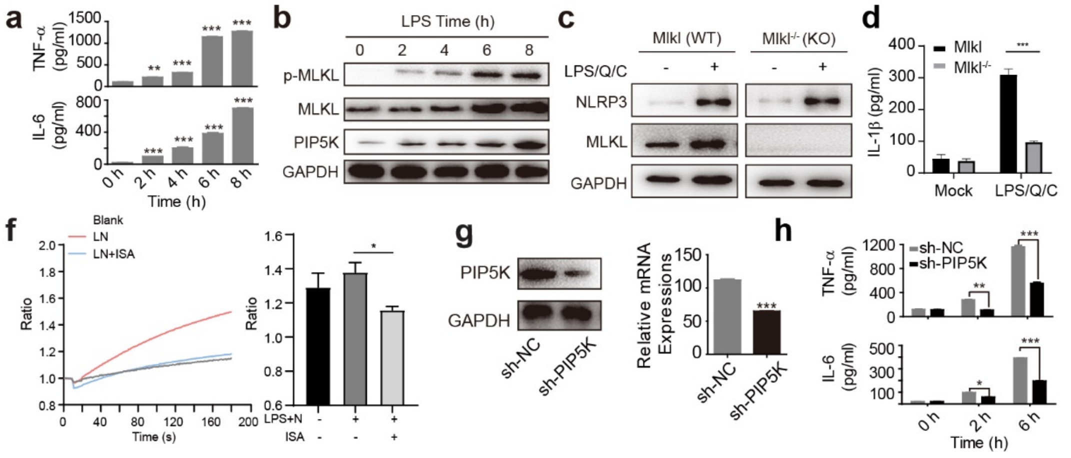

Supplementary Figure S9 Inflammation and protein analysis were obtained from BV2 cells after LPS treatment in different time points. a Cytokine analysis of IL-6 and TNF-α secretion levels by ELISA assay. **b** MLKL and PIP5K expression increased in LPS-treated BV2 cells without cell death. **c** The protein expression level of NLRP3 and MLKL in BMDM cells treated with LPS (10 ng/mL) plus 10 nM Smac-mimetic Compound A (C) and 20 μM Q-VD-OPh (Q). **d** Supernatants were assayed for IL-1β levels by ELISA. **f** Detection of K^+ efflux level of BV2 cells treated with LN (10 ng/mL LPS +10 μM Nigericin) with or without ISA ($n \geq 3$) (Left). Statistical analysis results were shown (Right). **g** BV2 cells stably expressing shRNA-NC or PIP5K-specific knockdown shRNA, respectively, were subject to western blotting and qRT-PCR analysis to determine PIP5K protein and mRNA levels. **h** Effect of $PI(4,5)P_2$ on LPS-induced cytokine production in BV2 cells. BV2 cells with downregulated $PI(4,5)P_2$ levels by PIP5K knockdown were treated with 100 ng/mL LPS (Left).*$P < 0.05$; **$P < 0.01$; ***$P < 0.001$; unpaired Student's *t*-test. All error bars are SEM.

Supplementary Table S1

Proteome data of the BV2 cells with or without LPS treatment, the area values under the curve (AUC) are shown for protein quantification.
